# Immune checkpoint inhibitor-induced AMPA2 antibody-positive encephalitis: a case report

**DOI:** 10.3389/fonc.2025.1684010

**Published:** 2026-01-21

**Authors:** Jing Li, Yanlan Huang, Jiemei Ye, Shaozhang Zhou, Yun Zhao

**Affiliations:** 1 Department of Neurology and Cardiology, Guangxi Medical University Cancer Hospital, Nanning, China; 2 Department of Neurology, Wuzhou Red Cross Hospital, Wuzhou, China; 3 Department of Respiratory Oncology, Guangxi Medical University Cancer Hospital, Nanning, China

**Keywords:** anti-AMPA2 antibody, autoimmune encephalitis, immunosuppressant, small-cell lung cancer, tislelizumab

## Abstract

Immune checkpoint inhibitors (ICIs) have revolutionized cancer treatment but are associated with a range of immune-related adverse events, including neurological complications. We report the case of a 64-year-old man diagnosed with small-cell lung cancer who developed AMPA2 antibody-positive autoimmune encephalitis after receiving tislelizumab combined with chemotherapy. The patient presented with a 3-week history of cognitive decline following the second cycle of treatment. Extensive testing confirmed the presence of a-amino-3-hydroxy-5-methyl-4-isoxazolepropionic acid 2 (AMPA2) antibodies in the cerebrospinal fluid and serum. The patient was treated with corticosteroids and intravenous immunoglobulin, resulting in significant improvement within 1 month. This case highlights the potential of ICIs to induce autoimmune encephalitis, which is a rare but serious complication. This finding underscores the importance of early recognition and prompt intervention in patients undergoing immunotherapy.

## 1 Introduction

Immune checkpoint inhibitors (ICIs) have achieved satisfactory therapeutic effects; however, with an increased understanding of these agents, complex and extensive immune-related toxicities have also been reported (1). ICIs have markedly improved therapeutic outcomes for a broad spectrum of malignant tumors, yet they are concomitantly associated with immune-related adverse events (irAEs) involving the nervous system. Notably, immune-related encephalitis represents a relatively rare yet potentially life-threatening neurological toxicity induced by ICIs. Due to its non-specific clinical manifestations, this condition is highly susceptible to a misdiagnosis as a neurological infection, metabolic disturbance, or central nervous system (CNS) tumor infiltration, which frequently leads to diagnostic errors and omissions, and thus, delays the administration of optimal therapy. The mechanisms underlying ICI-related encephalitis are currently understood as follows (2–4):

1. targeted tissue injury mediated by specific antibodies, such as the AMPA2 antibody-driven encephalitis in this case;
2. glial cell damage resulting from cellular immune activation; and
3. synergistic toxicity of combined drug regimens, which triggers pathological changes like blood–brain barrier (BBB) disruption.

The objectives of this case report are to present the specific clinical manifestations and share the corresponding treatment insights of this disease entity.

## 2 Case description

**Figure 1:**
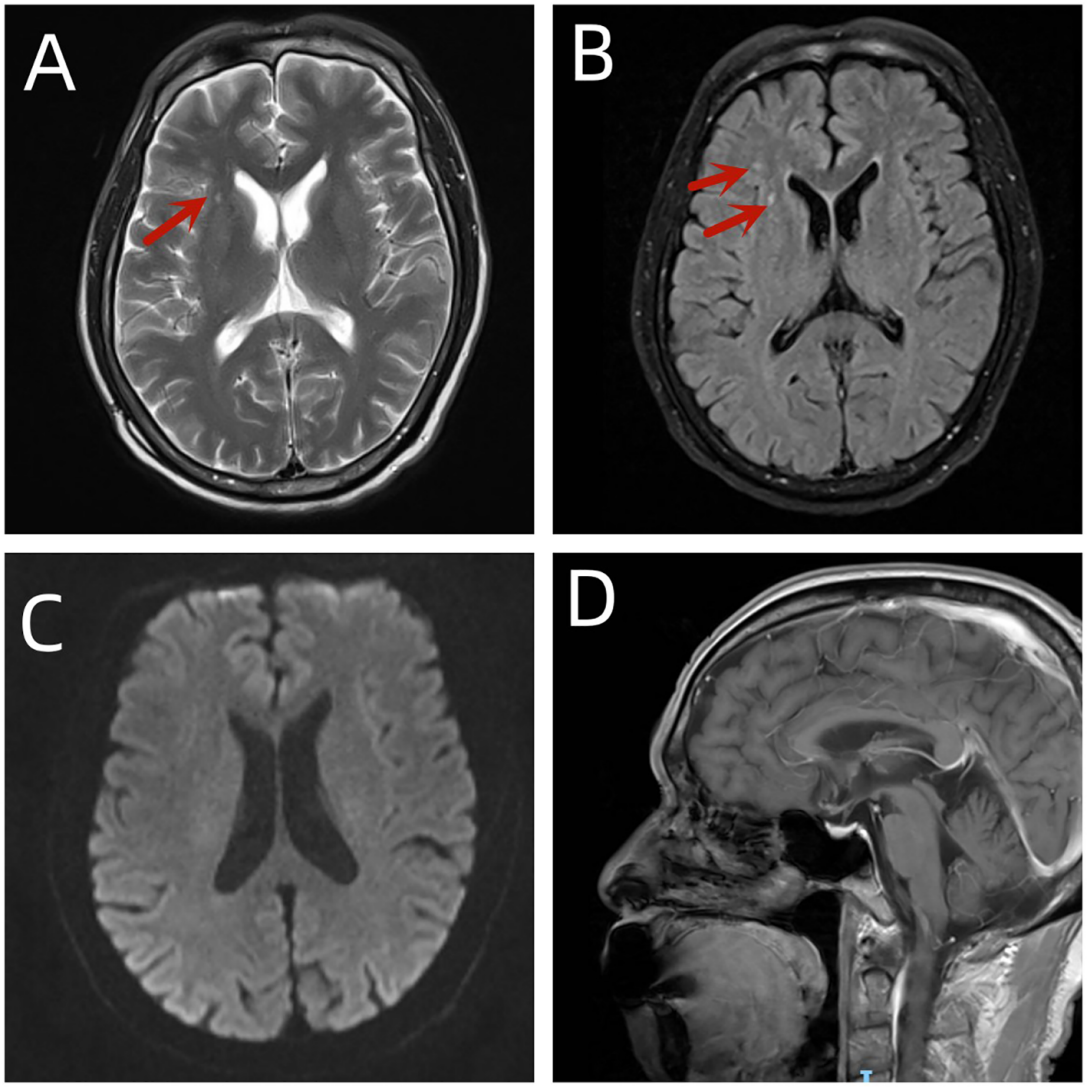
Four MRI brain scans labeled **(A–D)**. **(A)** Axial view showing a bright area indicated by an arrow. **(B)** Axial view with two arrows highlighting affected areas. **(C)** Axial view without annotations, showing symmetrical brain structures. **(D)** Sagittal view displaying the brain's side profile, detailing structures like the cerebellum and brainstem.

A 64-year-old man presented with a 3-week history of sudden cognitive decline. In March 2023, the patient was diagnosed with small-cell lung cancer on the right side, classified as stage IV, with T4N3M1a characteristics, indicating an advanced and widespread form of the disease. No neurological deficits were observed during physical examination. The first and second cycles of immunochemotherapy (tislelizumab + etoposide + cisplatin) were administered on 16 March 2023 and 12 April 2023, respectively. Three weeks after the second round of chemotherapy and immunotherapy, specifically tislelizumab combined with etoposide and cisplatin, the patient exhibited memory loss and impaired calculation abilities, evidenced by his inability to recall recent actions and to repeat tasks constantly. The patient had no history of hypertension or diabetes mellitus. There was no history of surgery or trauma. The cognitive function scales [Mini-Mental State Examination (MMSE) and Montreal Cognitive Assessment (MoCA)] revealed a decline in higher cortical functions, such as memory, calculation, orientation, and executive function. The rest of the nervous system showed no obvious abnormalities, and routine blood, stool, and urine tests were normal. Liver and kidney functions, blood lipid levels, thyroid function, blood glucose levels, and glycation were normal. The patient tested negative for specific T-cell detection for tuberculosis infection (T-SPOT), Epstein–Barr virus (EB), upper respiratory virus, SARS-CoV-2, and preoperative infections. Procalcitonin and C-reactive protein levels were normal. Tumor markers were negative. The anti-glomerular basement membrane antibody test was negative, the extractable nuclear antigen (ENA) antibody profile was negative, the double-stranded DNA quantification was normal, the anticardiolipin antibody was negative, and the immune panel was normal. The MMSE score was 10 (orientation score, 5 points; memory score, 1 point; and language ability score, 4 points), indicating moderate cognitive impairment (the lower the score, the more severe the loss of cognitive function). The MoCA score was 8 (visuospatial/executive score, 1 point; naming score, 1 point; attention score, 1 point; language score, 2 points; and orientation score, 3 points), indicating serious cognitive impairment (the lower the score, the more severe the loss of cognitive function). The Self-rating Depression Scale (SDS) score was 34, indicating a normal level (the higher the score, the more severe the situation). Finally, at admission, the Self-rating Anxiety Scale (SAS) score was 48, indicating a normal level (the higher the score, the more severe the situation). Residual urine, blood pressure in the supine position, and electrocardiographic findings were normal. Brain magnetic resonance imaging (plain and enhanced scans) showed no disease-related changes and only small ischemic lesions in the bilateral corona radiata and basal ganglia. These changes were considered non-specific (chronic infarct) (**Figure 1**). Chest computed tomography (CT) revealed a right hilar mass highly suspicious for central lung cancer, accompanied by multiple metastases involving the right lung parenchyma, right pleura, right supraclavicular fossa, mediastinum, and right hilar lymph nodes. Electroencephalogram findings were moderately abnormal (widely diffusely distributed slow waves were predominant). No abnormal spike waves, sharp waves, or three-phase waves were observed. Cerebrospinal fluid pressure in the left lateral position was 130 mmH_2_O (normal range: 80–180 mmH_2_O), white blood cell count was 5 × 10^6^/L, total protein was 0.56 g/L, glucose levels were normal, and chloride levels were normal (all data were from Guangxi Medical University Cancer Hospital). Tests for bacteria and *Cryptococcus neoformans* were negative. Aβ1-40, Aβ1-42, P-Tau181, and P-Tau were all normal. Exfoliative cytology of the cerebrospinal fluid revealed no cells of a different sex. Paraneoplastic antibodies in the cerebrospinal fluid and serum were negative. Cerebrospinal fluid viral metagenomic testing was negative. The Thermo Fisher EVOS M5000 cell imaging system was used to detect autoantibodies of autoimmune encephalitis using a cytometric bead array (CBA). Cerebrospinal fluid autoimmune encephalitis antibody status was as follows: anti-glutamate receptor (AMPA2) antibody IgG(+), 1:100. Peripheral blood analysis revealed the presence of anti-glutamate receptor (AMPA2) antibody IgG at a titer of 1:100; N-methyl-D-aspartate receptor (NMDAR), gamma-aminobutyric acid B receptor antibody (GABABR), and contactin-associated protein-like 2 (CASPR2) and other 16 antibodies were negative. Agarose gel electrophoresis (AGE) combined with the immunofixation technique detected positive results for oligoclonal bands. All reagents were purchased from KingMed Diagnostics. The pathology was determined as small-cell lung cancer.

The patient was diagnosed with ICI-associated autoimmune encephalitis with positive AMPA2 antibodies in Nanning, China.

### 2.1 Treatment and follow-up

**Figure 2:**
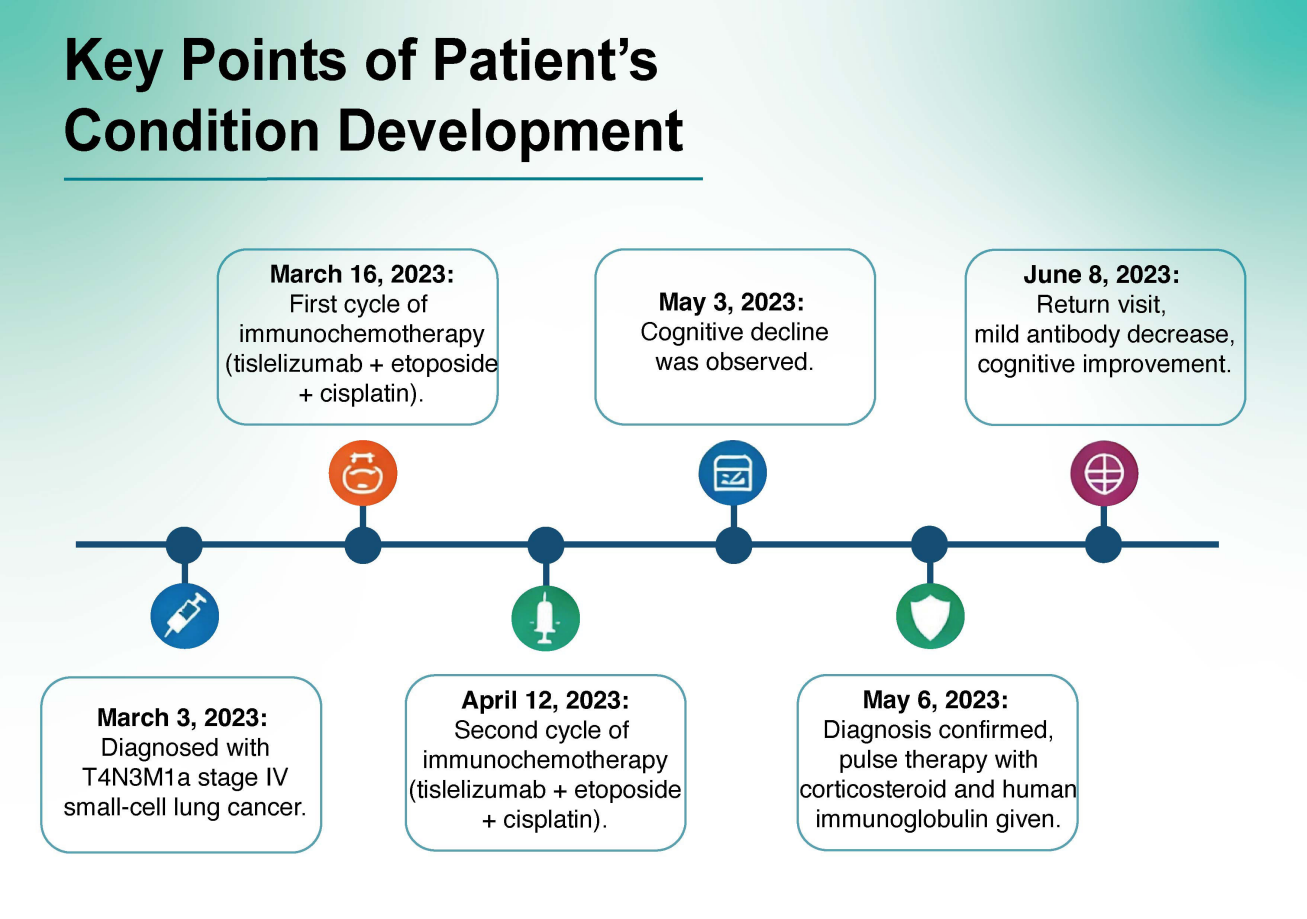
Key points of the development of the patient’s condition.

Per the criteria outlined in CTCAE v6.0, this adverse event is categorized as grade 3. Immune checkpoint inhibitor treatment should be immediately discontinued following confirmation of this diagnosis. Methylprednisolone was administered at 500 mg via intravenous infusion pulse therapy, with the dose halved every 3 days until it reached 60 mg, which was administered orally and reduced by 5 mg every 2 weeks. Human immunoglobulin was administered at a dose of 0.4 g/kg/day as continuous pulse therapy over 5 days. After 1 month, the patient’s cognitive function was assessed using the MMSE, resulting in a score of 15 (orientation score, 6 points; memory score, 2 points; calculation score, 2 points; recall score, 1 point; and language ability score, 4 points), and the MoCA, which yielded a score of 13 (visuospatial/executive score, 3 points; naming score, 1 point; attention score, 2 points; language score, 2 points; and orientation score, 5 points). Cerebrospinal fluid autoimmune encephalitis antibody status was anti-glutamate receptor (AMPA2) antibody IgG(+), 1:10. Peripheral blood analysis revealed the presence of anti-glutamate receptor (AMPA2) antibody IgG at a titer of 1:10. After 3 months, the MMSE score was 23 points (orientation score, 8 points; memory score, 3 points; calculation score, 4 points; recall score, 2 points; and language ability score, 6 points), and the MoCA score was 20 points (visuospatial/executive score, 4 points; naming score, 2 points; attention score, 4 points; language score, 2 point; abstraction, 1 point; recall score, 2 points; and orientation score, 5 points). Cognitive function exhibited a marked improvement compared with that observed 1 month prior, while antibody levels showed a moderate decline (**Figure 2**).

## 3 Discussion

ICIs disrupt immune homeostasis and induce diverse irAEs, with an overall incidence of 66%–72% (3). These toxicities typically involve the skin, intestines, lungs, liver, and endocrine organs. ICI-associated neurotoxicity constitutes 1.0%–12.0% of all reported irAEs and is more common in patients treated with anti-PD-1/anti-cytotoxic T-lymphocyte-associated protein 4 (CTLA-4) antibody combinations. Anti-PD-1 antibodies are associated with a lower risk of neurological irAEs compared with anti-CTLA-4 antibodies, a trend that extends to irAEs affecting other organ systems as well (2). Patients with ICI-induced encephalitis (ICI-iE) frequently manifest severe consciousness impairment, the rate of epileptic seizures is markedly lower than in the anti-LGI1 encephalitis group, and the oligonucleotide clones were negative. Partial symptomatic overlaps are prominent between ICI-iE and herpes simplex virus type 1 (HSV-1) encephalitis, which suggests that distinguishing these conditions clinically by symptoms alone is difficult (4). Positive oligoclonal bands were identified in this case, which is inconsistent with previous studies. This result might be associated with the testing approach used, or it could point to a specific type of encephalitis. Therefore, additional samples and rigorous, well-designed research are required to clarify this association. Studies have demonstrated that the most prevalent antibodies associated with ICI-induced toxic encephalitis include NMDAR antibodies, CASPR2 antibodies, neural endothelial antigen antibodies, and glial fibrillary acidic protein (GFAP) antibodies, among others. Despite the presence of these autoantibodies, most affected patients did not present with typical neurological syndromes. Furthermore, no definitive correlation has been identified between specific tumor types, ICI drug classes, and the induction of these brain-reactive antibodies (5). AMPAR (α-amino-3-hydroxy-5-methyl-4-isoazole propionic acid receptor) is a glutamate receptor that mediates synaptic plasticity and regulates the homeostasis of synapses. It is involved in learning and memory (6). AMPA2 antibody-positive autoimmune encephalitis caused by ICIs has not been previously reported. In this case, the brain MRI showed no obvious encephalitis, and the cerebrospinal fluid pressure was normal, with slightly higher protein levels. The main clinical manifestation was cognitive decline, which may be related to AMPA2 antibody positivity. The main clinical manifestations of anti-AMPAR encephalitis are symptoms of limbic encephalitis, with cognitive decline being the most common (7), and sudden short-term memory loss is the most common symptom (8), which can lead to orientation and executive dysfunctions and sometimes may be accompanied by epilepsy (9). As the AMPA-Ab titer decreased, the patient’s cognitive dysfunction gradually recovered. Similar recovery has been reported by Yu Jia et al. (8). Simultaneously, the prognosis of patients with anti-AMPAR encephalitis is related to the effective treatment of tumors, and regular tumor monitoring and treatment are necessary even after the neurological deficits have subsided (10). Controversy remains in existing case reports over whether positive AMPA2 antibody results are attributable to SCLC-associated paraneoplastic syndrome or ICI-induced encephalitis. For the patient in question, no neurological impairment was observed before symptom onset, while cognitive decline occurred explicitly after ICI treatment. No systemic tumor-related symptoms were present either. After suspending ICIs and implementing methylprednisolone pulse therapy, the patient’s symptoms resolved rapidly, and the antibody titer dropped accordingly. Thus, ICI-induced encephalitis is deemed to be the most likely diagnosis. Cognitive dysfunction in older patients with cancer is frequently overlooked in clinical practice. This case report aims to improve the understanding of the diversity of ICI-induced encephalitis. As the number of indications for these drugs increases, more patients will be exposed to them, and side effects are expected to become more frequent. Therefore, further research in this area is needed. Determining patients who are more likely to experience adverse immune events to prevent these side effects is required.

## Data Availability

The original contributions presented in the study are included in the article/supplementary material, further inquiries can be directed to the corresponding author/s.
